# Mapping the global distribution of coastal World Heritage Sites: Glo-CoH, a new Global Coastal Heritage dataset

**DOI:** 10.1038/s41597-026-07758-3

**Published:** 2026-07-24

**Authors:** David Bescoby, Joanne Clarke, Chukwuma Okolie, Robert J. Nicholls, Nicholas P. Simpson, Athanasios T. Vafeidis, Michalis Ioannis Vousdoukas

**Affiliations:** 1 School of Environmental Sciences, University of East Anglia, Norwich, UK; 2 Department of Archaeology, Durham University, Durham, UK; 3 School of History and Art History, University of East Anglia, Norwich, UK; 4 African Centre for Cities, University of Cape Town, Cape Town, South Africa; 5 Tyndall Centre for Climate Change Research, University of East Anglia, Norwich, UK; 6 School of Engineering, University of Southampton, Southampton, UK; 7 Climate Risk Lab, African Climate and Development Initiative, University of Cape Town, Cape Town, South Africa; 8 African Synthesis Centre for Climate Change, Environment and Development (ASCEND), University of Cape Town, Cape Town, South Africa; 9 Climate and Sustainability Programme, ODI Global, London, UK; 10 Department of Geography, Coastal Risks and Sea-Level Rise Research Group, Kiel University, Kiel, Germany; 11 Department of Marine Sciences, University of the Aegean, Mytilene, Greece

## Abstract

Coastal heritage sites face significant global challenges such as flooding and coastal erosion, exacerbated by climate-induced sea-level rise and coastal development. The ability to accurately simulate and assess potential exposure of heritage sites to these hazards has been hampered by a lack of digital high resolution spatial data accurately recording the topographic boundaries of heritage sites. The Glo-CoH dataset contains digitized boundary extents of all the heritage sites inscribed by the United Nations Educational, Scientific and Cultural Organization (UNESCO) as World Heritage properties of Outstanding Universal Value that are situated along the global coastline under 20 m Above Mean Sea Level (AMSL). It comprises a total of 1,250 individual serial sites covering the breadth of designated cultural, natural and mixed heritage sites and encompasses an area covering over 235 million hectares. A programme of site digitisation utilised extant site plans and Google Earth worldwide satellite imagery to locate and digitise heritage site boundaries. The quality of the resulting dataset was assessed by applying a mixed qualitative/quantitative validation procedure to a stratified sub-sample, encompassing the quality of source material, site identification, visibility and digitising accuracy. The validation procedure suggests 92% of digitized sites obtain validation scores indicative of optimal digitisation accuracy. The Glo-CoH dataset is made available for geo-spatial analysis and intended for use by researchers and practitioners looking to assess or plan environmental, climatic, economic, infrastructural and societal dimensions relating to coastal heritage at site, city, and regions up to global scales.

## Background & Summary

Between January 2022 and April 2024, the Global Coastal Heritage Mapping Project conducted a two-year long process of digitising the spatial extent of UNESCO coastal World Heritage Sites up to and including 2023 inscriptions (UNESCO, 2025)^1^. The resulting Global Coastal Heritage (Glo-CoH) dataset, Version 2 is designed to support the assessment of coastal flood and erosion risks including the effect of climate-induced sea-level rise. In addition, other coastal applications of the data can be considered. There currently exists no global scale high-resolution digital dataset of coastal heritage site boundary extents, limiting the spatial precision of modelled sea-level rise estimates (e.g. Marzeion & Levermann)^2^.

The project considered coastal sites located at or under 20 m Above Mean Sea Level (AMSL) - the Extended Low-Elevation Coastal Zone or E-LECZ (e.g. Wolff *et al*.)^3^. The E-LECZ encompasses all areas potentially affected by coastal erosion and flood hazards, including sea-level rise. Note that hydrological connectivity was considered at global scales so low-lying basins such as the Caspian and Dead Sea Basins were excluded from the dataset. However detailed hydrological connectivity within basins was not considered and users need to consider this factor in their analysis. The dataset comprises a total of 1,250 individual sites encompassing an area over 235 million hectares.

**Fig. 1:**
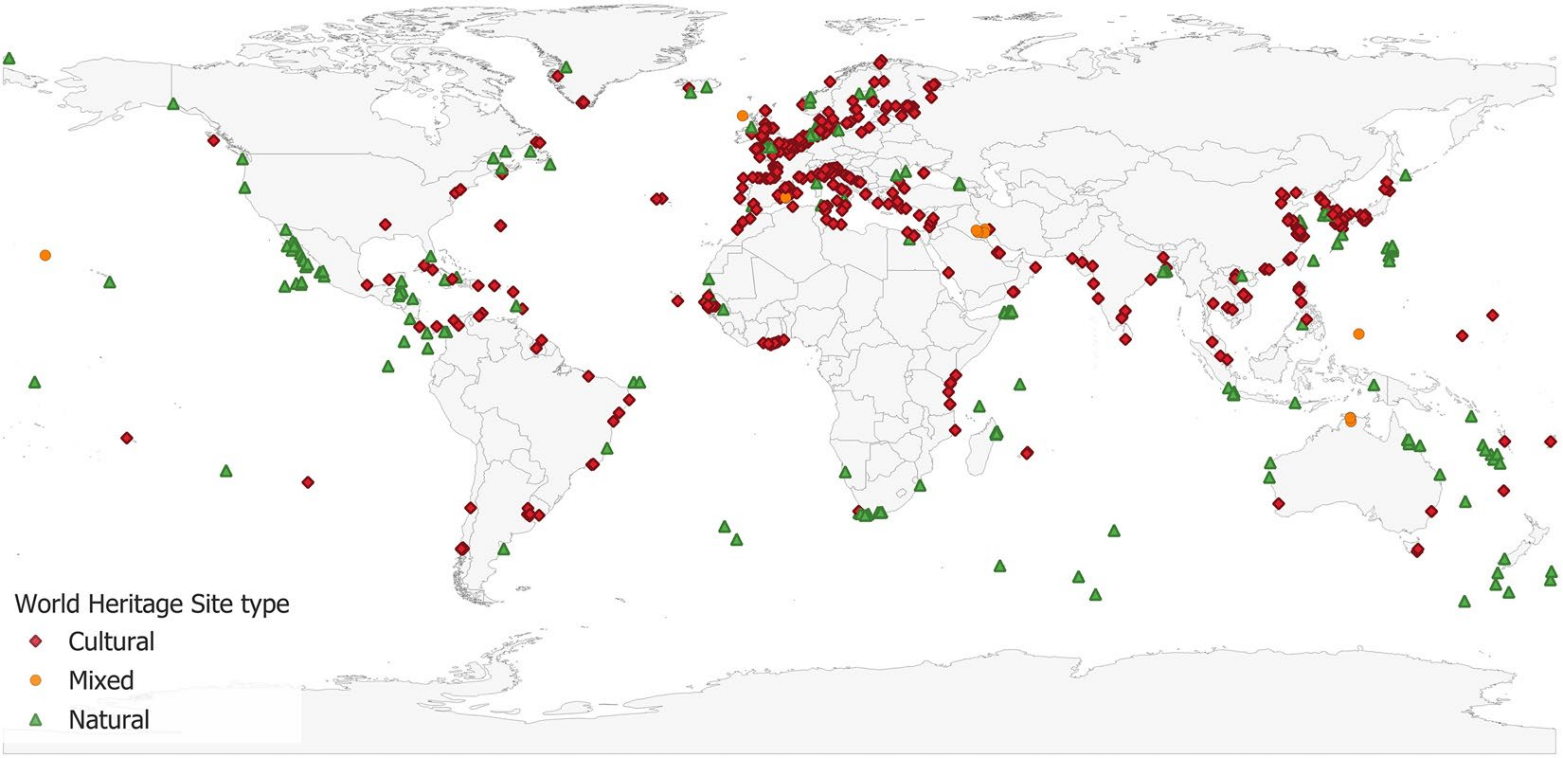
Global distribution of world heritage sites digitized by the Global Coastal Heritage Mapping Project.

At the end of 2023, there were 1,199 UNESCO World Heritage Sites listed in total - heritage that is recognized to have Outstanding Universal Value, of which 386 sites include elements sitting at an elevation at or below 20 m AMSL (UNESCO, 2025)^1^. These sites reflect a collective commitment to safeguarding the planet’s most precious coastal places for future generations (see Di Giovine, 2014)^4^. World Heritage Sites designated by UNESCO are further categorised as ‘Cultural’, ‘Natural’ or ‘Mixed’ (see Leask & Fyall)^5^; the presented dataset contains 289 cultural sites, 91 natural sites and 6 mixed sites. While many of these low-lying sites consist of a single site boundary, many also encompass multiple component parts. Within the Glo-CoH dataset, Version 2 there are 234 single element site designations, with the remaining comprising of component parts resulting in a total of 1,250 heritage sites within the dataset - the global distribution of these sites is shown in Fig. 1.

The publication of this dataset comes at a time of increasing interest in the resilience of global heritage against a backdrop of impacts from often rapidly changing environmental conditions. Recent global studies of this type include promoting sustainable development of cultural heritage sites in the face of land-cover change^6^, the risk global climate change poses to natural world heritage sites^7^ and assessing the vulnerability and resilience of UNESCO world heritage to global climate change^8,9^. To date, comprehensive digitized versions of World Heritage Sites have not been publicly available and we and other scholars have created our own geo-spatial datasets in order to undertake spatial modelling of hazards and risks to sites.

The presented dataset aims to facilitate the global and site level quantitative assessment of exposure of all coastal World Heritage Sites to the impacts of extreme sea levels and sea-level rise resulting from climate change. Such assessments will further quantify these risks, raising awareness of the urgent need for heritage adaptation and preservation in the face of climate change driven processes and ultimately minimising potential losses and damages to heritage assets.

Cultural heritage includes natural, cultural (tangible) and cultural (intangible), or mixed natural or cultural sites. Natural World Heritage Sites are areas of stunning natural beauty, which contain unique ecosystems, geodiversity and rare species. Sites are classified as mixed if they are of both natural and cultural importance.

## Methods

The dataset relies on published UNESCO maps submitted as part of the inscription process as the primary data source and on remotely sensed worldwide satellite imagery and terrain data available via the web and computer program Google Earth (GE)^TM^ for the subsequent identification and digitisation of heritage site polygon boundaries^1^.

GE mapping superimposes satellite imagery, aerial photography and Geographic Information System (GIS) data from a myriad of sources onto a three-dimensional (3D) globe (Gorelick *et al*.)^10^. For much of the earth, GE generates a Digital Elevation Model (DEM) based primarily on data from NASA’s Shuttle Radar Topography Mission (SRTM GL1) 1 Arc-Second Global data (30 m resolution), augmented and refined with numerous, often regional, high resolution data sources icluding JAXA’s ALOS, TanDEM-X, ArcticDEM, GMTED2010 along with local LiDAR data to improve accuracy in specific, high-relief or polar regions – see Farr *et al*.^11^, Rochmadi (2023)^12^. GE mapping data has found wide applications among researchers and practitioners not only in spatial-temporal mapping, but also in the accuracy assessment and validation of maps and spatial data (e.g., Luo *et al*., 2014; Luo *et al*., 2018; Lesiv *et al*., 2018; Pulighe *et al*., 2015)^13–16^. Several studies have confirmed the increasing adoption of GE for geospatial validation tasks (e.g., Dorais and Cardille, 2011; Kaimaris *et al*. 2011; Yu *et al*., 2012)^17–19^. In a recent review of GE for archaeological and cultural heritage (ACH) applications, Luo *et al*.^14^ stated that “GE has sufficient horizontal positional accuracy for searching and locating ACH sites”. Numerous mapping projects have historically utilized GE as a source of regionally/globally distributed ground truth data (e.g., Potere *et al*., 2009; Bontemps *et al*., 2011)^20,21^.

The accuracy of GE derived elevation data was hard to quantify in absolute terms given the global extent of the dataset and subsequent variations in the source data used. Several studies have derived regional accuracy estimates from networks of known ground reference points, expressed in standard statistical measures of Root Mean Square Error (RMSE), Mean Error (ME) and Maximum Absolute Error (MAE). A study from northern Egypt in 2016 reported ME values of between 0.51 and 1.52 m (RMSE 1.85–5.69 m)^22^. A more recent study from the district of Kerala, India over a range of terrain types utilising 900 ground survey elevation points returned an RMSE of 2.84 m, comparing favourably with other DEMs (STRM DEM 3.82 m; Cartosat DEM 5.27 m; Alos Palsar DEM 3.66 m)^23^. Another comparative study with ASTER elevation data in Malasia found a strong correlation (R^2^ = 0.917)^24^.

### Site identification and digitizing protocol

**Fig. 2:**
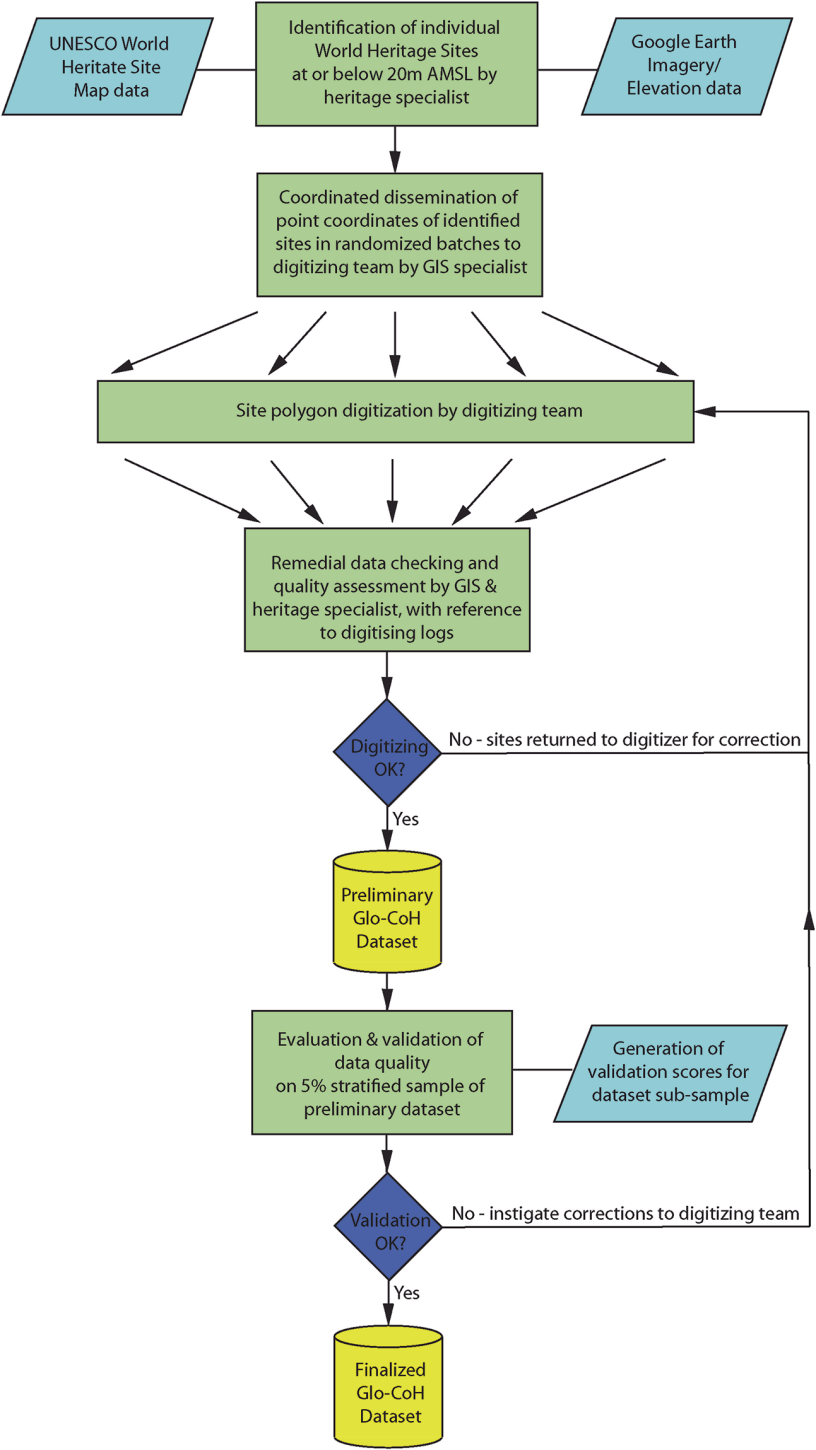
Flow diagram for the creation of the Glo-CoH dataset.

The scale of the digitizing programme necessitated the development of a digitizing protocol and associated training to allow a team of 14 digitizers from a range of backgrounds and locations to work on generating site boundary polygons in parallel. The protocol focused on the use of Google Earth Pro software as the primary digitising platform, providing straightforward access to regularly updated high resolution satellite imagery, as well as a catalogue of historical remotely sensed images. The workflow of the digitizing process is illustrated in Fig. 2.

The identification of coastal World Heritage Sites meeting the 20 m AMSL criteria was undertaken by a heritage specialist investigating the location of sites according to inscription plans, maps and spot coordinates published by UNESCO. The location and elevation of all coastal sites and their attendant component parts were manually verified by a heritage specialist with reference to GE imagery and DEM data to accurately locate sites and assess their spatial extent and elevation. Point coordinates were then generated for each identified site or site element and used by the digitizing team to initially locate sites flagged for digitization.

The digitization of corresponding heritage site polygons was overseen by a GIS specialist and individual digitizers were sent small, randomised batches of site coordinates and associated site information. Batches of digitized sites were then returned to the overseeing specialist for integration into the dataset following remedial quality control checks on site location, digitizing accuracy and resolution. Organizing the digitization process into an essentially hierarchical workflow, with a single heritage specialist responsible for identifying sites to be digitized ensured overall consistency in the selection of sites. Similarly, assigning digitizing and quality control oversight to one GIS coordinator generated a degree of uniformity in the resulting dataset. Finally, an evaluation and validation of data quality was undertaken on a 5% sample of the dataset by a second GIS specialist (described below).

### Digitizing process

Site polygons were digitized directly on Google Earth imagery, or by first overlaying georeferenced UNESCO site maps onto GE imagery. The nature and quality of available UNESCO maps was found to be highly variable and for maps without graticules/grids, georeferencing was undertaken using identifiable tie-points (matching reference points) from GE imagery. Site boundaries were delineated from the georeferenced maps, and/or from GE imagery - both map and GE representations were often combined to refine the digitized site boundary.

In general, sites were digitized at a scale of 1:1000, although this varied depending on the overall size and complexity of the site; smaller sites or complex areas of larger sites were commonly digitized at scales of 1:250. Only the actual site boundaries were digitized and no attempt was made to capture any associated site buffer zones, principally because there is no standardization of buffer zones for UNESCO World Heritage Sites – see UNESCO operational guidelines^25^ and Vanhuele & Vanneste (2025) for a fuller discussion^26^. Members of the digitizing team familiar with the nature of cultural heritage assets were able to make reflexive decisions around the most likely position of site boundaries when clearly visible in high resolution GE imagery, often resulting in more detailed/accurate site outlines than those depicted by the original UNESCO maps and site plans. Information associated with the digitized polygon feature and stored within the GIS (referred to in the text as ‘attribute data’) were standardized to include information relating to the identity and location of the site, following UNESCO Operational Guidelines – see Data Records below.

Each digitizer completed a digitizing log, allowing free form notes to be entered during the digitization of each site, noting the nature of the source mapping and any problems encountered, either in interpreting the mapping or identifying sites and boundaries within the GE Imagery. The digitizing log provided additional information useful in subsequent data quality assessment.

### Additional data sources

13 percent of digitized sites were derived or based on material from three existing digital data sources:

1. The African heritage sites digitized as part of an assessment of their exposure to coastal flooding and erosion along the African coastline (Vousdoukas *et al*.^27,28^.
2. Digital site data generated by Reimann *et al*.^29^ during their assessment of Mediterranean UNESCO World Heritage at risk from coastal flooding and erosion. The World Heritage Site datasets produced for this study are available in text format (CSV) and polygon vector format^30^.
3. Polygons for United Kingdom coastal sites sourced from Historic England’s Open Data Hub, available free under the Open Government Licence v3, © Historic England 2026, Contains Ordnance Survey data © Crown copyright and database right 2026^31^.

Pre-existing data being integrated into the new dataset were first evaluated with reference to the latest UNESCO World Heritage Site inscription (dossier) data, ensuring represented property boundaries were accurate and up to date. The spatial accuracy of data was then evaluated for site location, digitizing accuracy and resolution, in accordance with the digitising protocol outlined above. In a number of instances, adjustments to property boundaries represented in existing data were required to update property boundary extents or expand into multiple (discrete) component parts. Where the property appeared in multiple data sources and differences in the property polygon boundary existed, the most accurate boundary representation according to the above criteria was taken as the basis for integrating existing data into the new dataset. Table 1 summarises the remedial steps taken during the integration process. The associated polygon feature information (attribute data) was not taken from existing datasets, but were instead recorded afresh with reference to the latest UNESCO published data, to ensure accuracy and the standardization of information within the Glo-CoH dataset.

**Table 1:** Summary of UNESCO World Heritage Site data integrated from existing data sources.

Data source	Number of sites integrated	Number of sites modified	Nature of polygon modifications
African Heritage sites (from Vousdoukas *et al*.)^27,28^	89	15	UNESCO boundary updates; Property division into multiple component parts.
Mediterranean Heritage sites (from Reimann *et al*.)^29,30^	24	9	UNESCO boundary updates. Improved boundary resolution (5 instances).
Historic England^35^	44	2	Property division into multiple component parts.

The resulting dataset consists of 1,250 digitized polygons of heritage site boundaries at or below 20 m AMSL. The dataset contains polygons representing cultural (1,028), natural (204) and mixed (17) World Heritage Sites meeting the 20 m AMSL criteria. Individual component parts of composite World Heritage Sites meeting the 20 m AMSL criteria are treated as discrete entities.

## Data Records

The Glo-CoH dataset (Version 2) and all supporting data is available from ZivaHub at the University of Cape Town^31^. This replaces the original Version 1 of the dataset which ended in 2023.

The dataset is provided as GIS files in both Geopackage and Shapefile format:

- Global Coastal Heritage Dataset (Glo-CoH) - Version 2.gpkg
- Global Coastal Heritage Dataset (Glo-CoH) - Version 2.shp

Note that shapefile field names are restricted to 10 characters.

There is an accompanying Microsoft Excel spreadsheet containing all associated GIS metadata (attribute table) data.

Associated GIS metadata records (attribute data) contain nine field of data, consisting of 1) a unique site ID number, 2) the UNESCO site designation code (Site Designation), 3) the UNESCO designated name of the site or component part (Name of Site), 4) the overarching name of the deignated property (Designated Name), 5) The country containing the site (Country), 6) the country ISO code (ISO Code), 7) the UNESCO designated sub-region (UNESCO Designated Sub-Region), 8) the IPCC WGII Sub-Region (IPCC WGii Sub-Region and 9) the area of the site in hectares (Area (ha)).

## Technical Validation

A procedure allowing digitized property boundaries to be characterised and assessed with regard to ‘closeness of fit’ to corresponding site boundaries visible in GE imagery was developed, providing a means of evaluating a sample of the dataset. This evaluation process provided a means of validating a sample of the digitized data in respect to perceived polygon accuracy and of potentially identifying recurrent issues affecting the quality of the dataset.

The procedure was developed by the authors specifically as a quantitative means of scoring individual polygon ‘quality’, considering a range of factors encompassing the accuracy of the available source mapping; the current and historical visibility of site boundaries in GE imagery and the resulting spatial accuracy of digitized polygons. We felt that these three parameters; source data accuracy, site visibility and resulting digitising accuracy, effectively controlled the overall spatial accuracy of the resulting dataset. Once evaluated and combined into an overall score for each digitised polygon, a threshold value could then be set, whereby digitized polygons scoring below the threshold were flagged for further investigation, error characterization and remedial corrective measures. The evaluation/validation procedure was undertaken independently by a second GIS specialist and recorded using a standardized recording form, completed for each site evaluated. The results were then reviewed together with the project GIS specialist.

### Validation parameters

The resulting procedure encompassed both qualitative and quantitative evaluation to derive an overall score relating to data quality against the following criteria:

- Quality of Source Mapping. The mapping was evaluated true or false in regard to the following five parameters: 1) Map legend: the symbol (and colour) of the heritage site is visible and well represented on the legend. 2) Spatial reference: the presence of graticules or a coordinate grid to aid georeferencing of the map/plan. Also includes details of the map projection/datum. 3) Legibility: labels/annotations on the maps (e.g. site name, street names) are legible. 4) Site representation: the area boundary of the heritage site is well depicted and easily decipherable from the map. 5) Graphic quality: the map is of sufficient graphic quality to enable adequate understanding of the site location, and its relationship/association with adjacent landmarks. The five scores were totalled to give a qualitative score value between 0 and 5.
- Visibility of Property Features on GE imagery. Property boundaries are ranked on visibility and distinctiveness: 1 - very low, 2 - low, 3 - moderate, 4 - high and 5 - very high, resulting in a qualitative score between 1 and 5.
- Polygon accuracy. A quantitative measure of polygon accuracy was undertaken for each polygon by constructing buffer tramlines 5 m either side of the digitized polygon. A count was then made of the number of times the site boundary visible on GE fell outside the buffered area along the digitized polygon boundary. This was then divided by the polygon’s perimeter to provide an accuracy score (5 m Buffer Score). The latest GE imagery has an average spatial (positional) accuracy of c. 5 m – see above, so applying the above metric ensures that polygon positional accuracy (assuming correctly identified site boundary location) is no greater than c. 10 m. In cases where the digitized polygon deviated more significantly from the apparent site boundary visible in GE, or, when this was unclear from the source mapping, the length of the incorrect section of polygon was measured and similarly divided by the total perimeter to give an overall accuracy score. This overall approach seemed appropriate for the diverse range of polygon sizes within the dataset, which varied from 0.001 ha to 4,084,507 ha; counting instances of boundary lines falling outside the 5 m buffer proved appropriate for small sites, while measuring areas of deviation more so for larger sites.

The total score for an evaluated polygon was derived by adding the ‘Quality of Source Mapping’ and ‘Visibility of Site Features…’ scores and multiplying the result by the ‘5 m Buffer Score’ – see Eq. (1) below:

1 $$Score=(Quality\,of\,Source\,Mapping+Visibility\,of\,Property\,Features)\times \left(1,-,\frac{5m\,Buffer\,Score}{polygon\,perimeter\,(m)}\right)$$

The evaluation process also assessed the accuracy of the associated GIS data records (polygon attribute data) described above by checking entered text against the UNESCO property records published online.

### Sampling data for validation

A stratified sampling strategy was derived to ensure the large variance of polygon sizes (measured as enclosed area) was accounted for within the derived evaluation/validation sample (Lohr, 2010; Thompson, 2012)^32,33^. Two characteristics were chosen for stratification, ‘Polygon Area’ and geographical ‘sub-region’, which contains five categories. ‘Polygon Area’ categories were derived by establishing six size categories. The highly skewed nature of the distribution of polygon sizes led to a selection of six bins spanning unequal size ranges that captured ~208 sites in each. This scheme resulted in 30 sub-groups from which two sites were randomly sampled, giving a total sample size of 60 sites ~5% of the overall 1,250 sites and serial sites that make up the dataset. The only sampling stipulation was that samples from any one of the 30 sub-group bins should be from different countries (the exception being ‘Arab States’/ ‘0–0.3 ha’, where all sites are from Bahrain).

The evaluation sample of 60 sites was found to contain 46 (76.6%) cultural sites, 12 (20%) natural sites and 2 (3.3%) mixed sites – a distribution similar to that of the overall dataset.

**Fig. 3:**
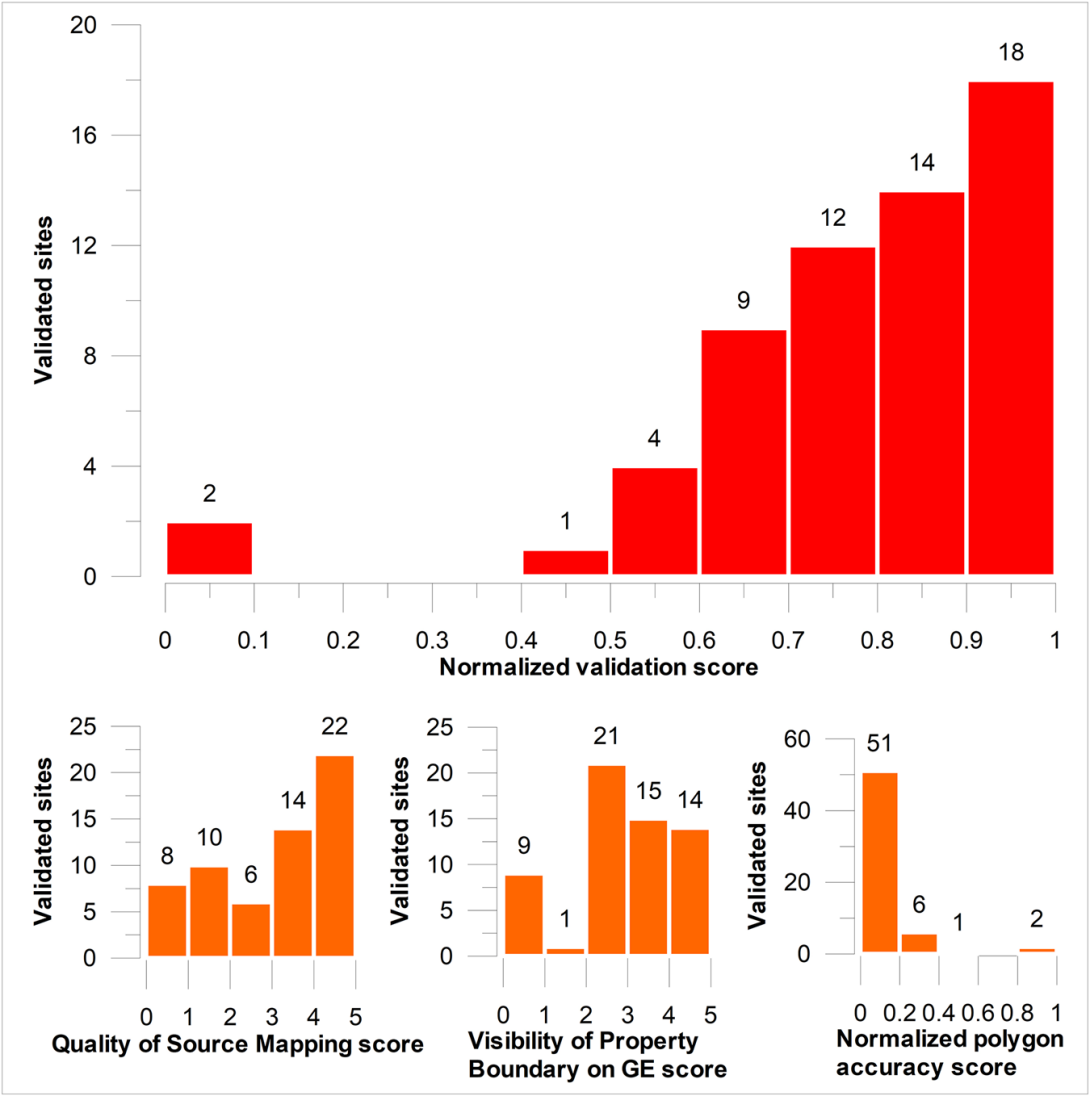
Histogram showing the distribution of normalised polygon validation scores for a stratified sub-sample of sites (red) and histograms of the ‘Quality of Source Mapping’, ‘Visibility of Property Boundary on GE’ and normalized polygon accuracy scores (orange).

**Fig. 4:**
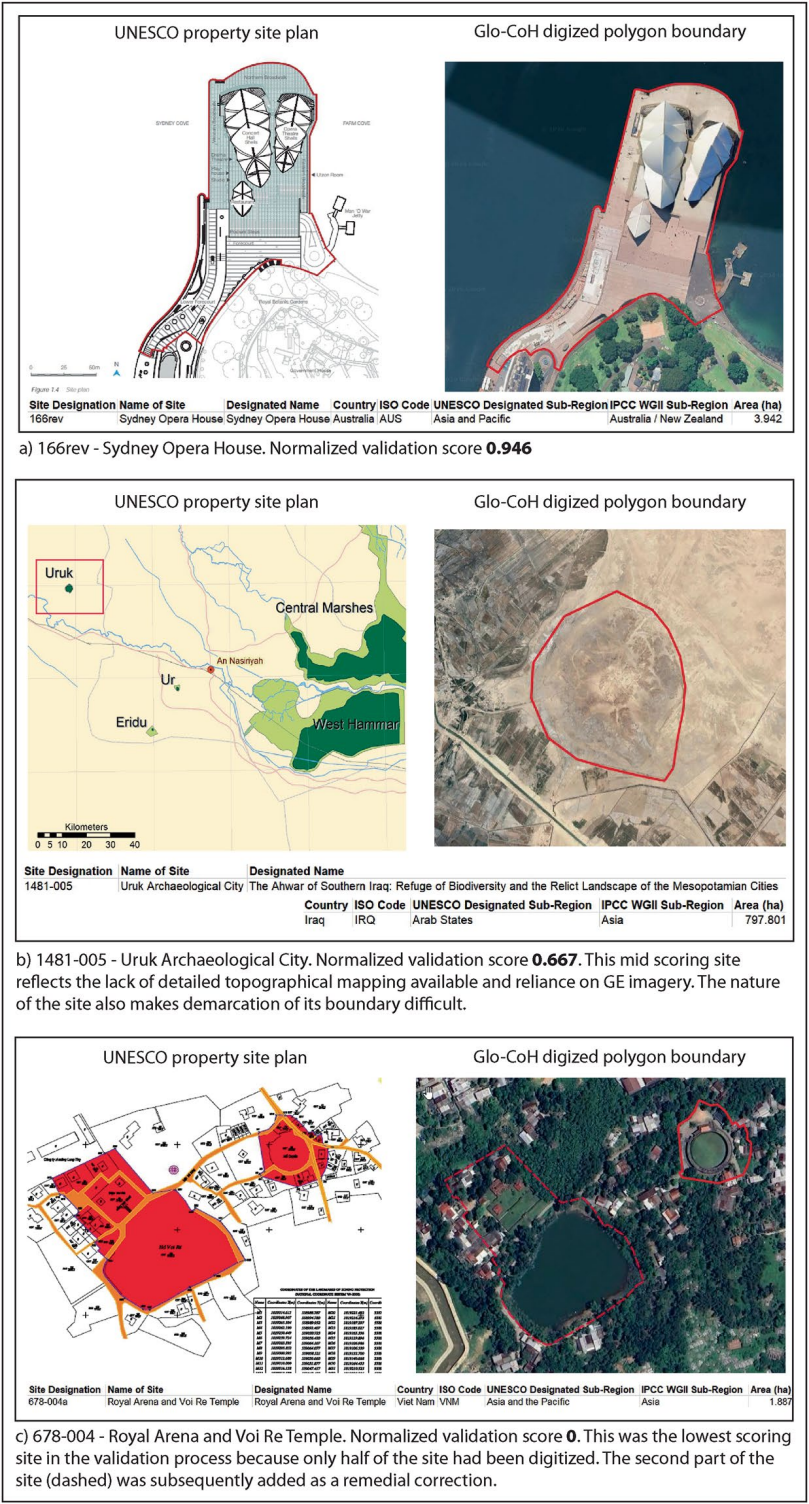
Three examples of evaluated polygons and their normalised validation scores, illustrated a high scoring site, a mid-scoring site and the lowest scoring site.

On evaluation of the 60 sampled sites, polygon scores were normalized and ranked in ascending order, as illustrated in Fig. 3, along with frequency distributions of the ‘Quality of Source Mapping’, ‘Visibility of Property Boundary on GE’ and normalized polygon accuracy scores. Illustrated examples of high, mid and low scoring sites are shown in Fig. 4. The 10 sites at the bottom of the ranking (scoring between 0 and 0.62) were further evaluated to determine the underlying cause of the low score and if any common factors were identifiable, such as site type, site size, geographical location or digitizer.

Of these 10 sites, the five lowest ranking sites were found to contain significant inaccuracies, such as mis-identification of the site to be digitized (2), partial digitization of the site (2) – see Fig. 4c, and poor replication of a site boundary (1). The five highest ranking of the 10 sites were considered to have been digitized as accurately as source mapping and visibility of salient features on GE allow. Four sites were additionally found to contain errors associated with their corresponding polygon attribute data stored within the GIS. The errors consisted of three mi-spelled property names, and one instances of an out of date ISO country code.

An estimate of overall accuracy, based on the evaluation of the 10 worst scoring sites in the validation sample, of which 5 sites were considered capable of remedial correction, suggests 8.3% of sites in the sample could be improved. With regard to the corresponding GIS attribute table data, 6.7% of the sites in the sample contained inaccuracies.

No common factors, such as polygon source/digitizer were found to correlate with poor polygon accuracy. Similarly, we applied a one-way analysis of variance (ANOVA) test to determine whether there was a significant difference between the mean validation scores from the five geographic regions, which returned a P-value of 0.357 (alpha = 0.05), indicating there is no significant difference between geographic regions.

## Usage Notes

The validation process described provided a means of assessing the overall uncertainty of the Glo-CoH dataset.

The mean average ‘Quality of Source Mapping’, reflecting the perceived overall quality of available UNESCO mapping was 3.47 (range 0–5) in the validation sample, with 37% of property mapping achieving a top scope of 5 – see Fig. 3. The average ‘Visibility of Property Features on GE’ score was 3.4 (range 1–5), with most (35%) falling into the ‘Moderate’ category. Both scores would appear to infer that overall, both the adequacy of the published UNESCO maps and the overall visibility of sites within GE imagery are sufficient for the effective digitization of property boundaries. However, once the site type (cultural, natural or mixed) is considered, the ‘Visibility of Property Features on GE’ score for natural sites drops to an average of 2.3, and for the two mixed sites, to an average of 2. This likely reflects the difficulties experienced by digitizers in accurately delineating the boundaries of these site types using satellite imagery. However, a one-way analysis of variance (ANOVA) test to determine whether there was a significant difference between the mean validation scores from the three site types returned a P-value of 0.717 (alpha = 0.05), indicating there is no significant difference in the overall validation scores of natural or mixed sites, compared with cultural sites. As noted above, not significant difference between validation scores and geographic region was evident.

The dataset was compiled specifically for modelling the impacts of climate driven sea-level rise. Consequently, some sites have been split into separate sub-sites where clearly delineated by coastal waters, e.g. island sites with associated islets or elements of an atoll.

The ongoing development of the Glo-CoH dataset is an iterative process, incorporating the periodic changes made by UNESCO to its dossier of inscribed properties. It is planned that the Glo-CoH dataset will be subject to a programme of regular (annual) review and revision, taking into account heritage sites that have been newly inscribed over the subsequent year, as well as alterations to existing inscriptions and the delisting of sites from the World Heritage List, or other revision as necessary.

There is also scope in our future research to extend the methodologies presented to incorporate Artificial Intelligence (AI) assisted tools in the process of identifying and delineating World Heritage Site property boundaries from a range of digital data sources. Geospatial AI (GeoAI), such as Meta’s Segment Anything Model (SAM) could well become appropriate tools for automating the recognition and tracing of polygon boundaries^34^. While we have focused our efforts on developing a costal heritage dataset, primarily to aid in the assessment of heritage assets at risk from climate-induced sea-level rise, it is conceivable that enhanced methodologies incorporating task automation using machine learning models would allow us to consider extending the dataset to include non-coastal UNESCO heritage properties.

### Dataset applications

One of the overall aims of the dataset was to provide heritage managers with a catalogue of up to date and accurate spatial boundaries for inscribed coastal world heritage properties, useful in the analysis of future environmental threats, such as flooding and erosion. As a publicly available dataset any heritage specialist or scientist with an interest in heritage sites will be able to use the dataset as the basis of their own analysis and modelling research at the global scale. We have endeavoured to make the dataset as user friendly as possible by using standard GIS file formats, which should allow users the flexibility to manipulate, convert and export the data in ways applicable to their application.

## Data Availability

Version 2 of the Glo-CoH dataset and all supporting data is available from ZivaHub at the University of Cape Town – 10.25375/uct.28547267. https://figshare.com/s/23e443449ab774999814.

## Code availability

No code was used in the creation of this dataset.
